# Development of a clinical tool for rating categories of the ICF Rehabilitation Set in Polish practice

**DOI:** 10.1038/s41598-023-28441-2

**Published:** 2023-01-24

**Authors:** Agnieszka Wiśniowska-Szurlej, Agnieszka Ćwirlej-Sozańska, Anna Wilmowska-Pietruszyńska, Bernard Sozański

**Affiliations:** 1 Institute of Health Sciences, Medical College of Rzeszow University, 35-310 Rzeszow, Poland; 2 Rehabilitation Center Donum Corde, Budy Głogowskie, 36-060 Głogów Małopolski, Poland; 3 Faculty of Medicine, Lazarski University, 02-662 Warsaw, Poland; 4 Institute of Medicine, Medical College of Rzeszow University, 35-310 Rzeszow, Poland

**Keywords:** Health care, Medical research

## Abstract

Rehabilitation is considered a key health strategy in the 21st century. The aim of rehabilitation is to optimize the functioning of patients. The International Classification of Functioning, Disability and Health (ICF) is a framework for describing and organizing information on functioning and disability. Current international efforts to implement ICF in rehabilitation practise include the implementation of ICF Core Sets and operationalize ICF tools for clinics. The aim of the study is to create simple, intuitive descriptions and an initial reference guide for the assessment of the ICF Rehabilitation Set in Polish practice. The development of the Polish version of ICF Rehabilitation Set involved the following steps: (1) identification of ICF Rehabilitation Set categories; (2) development simple, intuitive descriptions; (3) the drafting of the rating reference guide by a multidisciplinary panel following the process employed to develop the Japanese version. The Polish version of ICF Rehabilitation Set, the simple, intuitive descriptions for 29 categories and the rating reference guides were successfully developed. The Polish version of ICF Rehabilitation Set proposed by us is a reference framework for the harmonization of existing information on the functioning and disability of people participating in the rehabilitation process.

## Introduction

According to the World Health Organization (WHO) rehabilitation is defined as “a set of interventions designed to optimize functioning and reduce disability in individuals with health conditions in interaction with their environment”^1^. Rehabilitation is considered a key health strategy in the 21st century. Demographic and epidemiological trends indicate that human functioning is one of the most important indicators of population health, apart from morbidity and mortality. Thus, it suggests that the main focus of healthcare will be on the real health needs generated by long-term treatment of chronic diseases, including comprehensive enhancement and intensification of rehabilitation^1,2^.

The aim of rehabilitation is to optimize functioning of patients. WHO indicates International Classifications and Terminologies including the International Statistical Classification of Diseases and Related Health Problems (ICD) and the International Classification of Functioning, Disability and Health (ICF) as the global standards for collecting data regarding health, clinical documentation and statistical data aggregation^3^. ICF and ICD provide health information in relation to diagnosis (ICD) as well as functioning and disability (ICF)^4^. In 2019, WHO launched an eleventh version of ICD (ICD-11), which was supplemented with a section presenting an assessment of functioning containing selected ICF categories^5^.

The ICF is based on a holistic biopsychosocial disability model that allows for a comprehensive and wide approach to the problems of an individual related to functioning in his /her living environment^6^. The model contains 6 components, including: (1) body functions which make up physiological and psychological functions of the body; (2) body structures that are its anatomical parts; (3) activity as performing actions or tasks by the individual; (4) participation meaning involvement in life situations; (5) environmental factors that constitute physical, social or attitudinal elements of the environment in which people live; (6) personal factors constituting the life background and the life situation of an individual, comprising the features not related to a health condition^7^. This approach provides a multifaceted and multidimensional view of human functioning. It also creates a useful framework for understanding the interaction between individual components of the biopsychosocial model.

The ICF was developed and approved in 2001 and many initiatives have been implemented since then^4,5,8,9^. In order to make the classification more suitable for everyday use, ICF Core Sets have been developed. The ICF Core Sets provide lists of core categories that are relevant to specific medical conditions and healthcare contexts^10^. The ICF Rehabilitation Set includes 30 ICF categories and is used in the context of describing functioning in various clinical populations among adults, at various stages of patient rehabilitation in inpatient and outpatient rehabilitation facilities, as well as in day care centres^11^. The rating system proposed in the ICF consists of so-called "qualifiers". They are used to code the severity of a functional problem. The qualifiers are as follows: 0 (0–4%), no problem; 1 (5–24%), mild problem; 2 (25–49%), moderate problem; 3 (50–95%), severe problem; 4 (96–100%), complete problem; 8, not specified; and 9, not applicable^7^. Environmental factors interact with all components of the biopsychosocial model, making a facilitating or hindering impact of physical and social world and attitudinal system. Qualifiers linked to the category of activity and participation, by determining the level of performance, make it possible to observe the extent to which environmental factors act as a barrier or a facilitator^7^.

The lack of detailed guidelines presenting how to use the qualifiers can make it difficult to assess the patients consistently. In order to solve this problem, it is important to collect the already existing data on the assessment of functioning by means of the ICF and to develop explanations for codes and qualifiers adapted to the clinical conditions in a given country.

The ICF as a classification is not an assessment tool in itself, but it is a framework based on a biopsychosocial model that enables a comprehensive description of functioning and disability of the examined person in the context of his/her life^12^. The Global Disability Action Plan (2014–2021) of the WHO recommends the use of the ICF as a superior component of the diagnosis and assessment related with the rehabilitation process^13^. The ICF is accepted by all the WHO Member States, but its use in clinical practice is still limited^14^. One of the reasons is the limited number of clinical tools that use language familiar to healthcare professionals while still following the ICF concept. Therefore, in order to facilitate the implementation of the ICF in daily practice, it is necessary to develop a universal and health worker-friendly assessment tool. Cooperative and uniform implementation of the ICF to the Polish healthcare system, as well as other public services, must be coordinated. It is very important to introduce a universal language that would allow us to transfer the information about the state of human functioning in healthcare systems. Consequently, the aim of the study is to create an initial reference guide for the assessment of the ICF Rehabilitation Set in Polish practice.

## Materials and methods

The development of the Polish version of ICF Rehabilitation Set involved the following steps: (1) verification of ICF categories; (2) development of simple, intuitive descriptions; (3) development of the first Polish version of the rating reference guide.

### Verification of ICF categories

The first stage in the verification of the ICF categories was to conduct a discussion panel consisting of rehabilitation experts with a minimum of 3 years of work experience in an inpatient rehabilitation department in a hospital or a rehabilitation centre, a day rehabilitation centre or an outpatient rehabilitation facility. The members of the discussion panel included: a medical rehabilitation physician, a primary care physician, physiotherapists, an occupational therapist, a psychologist, a speech therapist, a nurse, a social worker and members of the Polish Council for the ICF. Specialists were recruited from inpatient and outpatient rehabilitation centres in the country. Medical facilities conducting rehabilitation were drawn from the register of medical facilities. The inclusion criteria for these units was to have an expert in the field of rehabilitation with at least 3 years of experience at work with the patient. If the centre refused to participate in the study or did not have an expert with the above-mentioned experience, another centre was drawn from the pool.

The task of specialists was to assess the ICF categories in terms of their importance for rehabilitation. The expert survey was carried out in the form of an online questionnaire. This expert survey, consisted of open questions and the answers to the questions regarding the most common problems, barriers and facilitations of people participating in the rehabilitation process, were identified, distinguished and linked to the ICF. Selected categories were counted only once per expert. It was assumed that the ICF categories, which were considered very important by at least 3 experts, were included in the list of categories qualified for the next stage of verification.

The second step aims were to identify the most common problems experienced by patient with health condition that was documented in a clinical setting. The discussion panel was attended by 21 adults and older people. People participating in rehabilitation at the Donum Corde Rehabilitation Centre were included in the mentioned group. The group of 21 patients included 13 women and 8 men, their mean age was 45.8 ± 13.4 years. The reasons for participation in inpatient rehabilitation were: cardiological (n = 4), neurological (n = 8), pulmonary (n = 3) and orthopaedic diseases (n = 6).

People participating in rehabilitation took part in interviews carried out by physiotherapists. The interviews aimed to identify the most common problems with functioning related with the current state of health, recorded in a clinical setting. During the interviews, the subjects determined aspects specific to the rehabilitation process and a given health condition that were important to provide a comprehensive description of their functioning. ICF categories that were considered a problem, barrier or facilitator were included in the candidate categories. Interview protocols were developed by the principal investigator, supplemented with relevant medical data contained in patients' medical records relevant to ICD-10. During the analysis process, the physiotherapist also discussed the patients' problems in an interdisciplinary team consisting of: a physician, physiotherapist, nurse, pharmacist, occupational therapist, speech therapist and psychologist to determine their relationship with ICF. Categories that were identified as a problem, barrier or facilitator by at least 75% of interviewees were included in the list of proposed ICF categories.

The methodology of the discussion panel was compliant with the WHO guidelines^15^ and was also based on the experience of other researchers^16,17^.

The final verification of the ICF categories proposed for inclusion in the Polish version of ICF Rehabilitation Set was carried out by a research team consisting of 4 persons with the title of associate professor and PhD. All researchers had experience in the use of the ICF classification in scientific research and clinical practice and they dealt with treatment, rehabilitation, education or medical statistics. The team analysed the obtained proposed ICF categories from the stage of experts (in the field of rehabilitation) examination as well as patients participating in physical rehabilitation. Individual categories from the ICF Rehabilitation Set were considered confirmed if the same category emerged in the course of interviews with patients and a group of rehabilitation experts. In consequence, three groups of categories have been identified: (1) ICF categories identified as relevant by patients and experts, hence represented in the ICF Rehabilitation Set, (2) ICF categories that were represented in the ICF Rehabilitation Set but not identified as important by the discussion panel, and (3) ICF categories that were considered important by the members of the discussion panel, but were not included in the ICF Rehabilitation Set^18^. The ultimate list of ICF categories consisted of codes that were considered relevant by at least 75% of the participants of the discussion panel.

### Development of simple, intuitive descriptions

To develop simple, intuitive descriptions of the Polish ICF Rehabilitation Set based on the original ICF category descriptions included in the classification, a consensus conference with multidisciplinary rehabilitation experts was conducted.

The consensus process included 3 groups of 7 experts with experience in clinical work with the patient. In each group, one moderator and an assistant for the ICF implementation team in Poland were appointed. The assistant was responsible for taking notes during the conference, but did not have the right to vote. Each group received initial suggestions for simple, intuitive descriptions of each ICF category. Descriptions were prepared on the basis of the results of previous conferences in Japan^19^, Italy^15^ and China^20^. The participants were asked to read and discuss the initial suggestions for descriptions. Then, each participant voted on whether the description was simple and intuitive enough to be used in everyday clinical practice. It was important to maintain the context of the original description of the ICF categories. During the first vote, consensus was achieved when the description of each ICF category reached 75% agreement in each group. The categories that did not reach consensus in the first vote were re-described, discussed and voted on again. A similar voting was conducted three times. The final versions of the ICF category descriptions were approved by the members of the Council for ICF Implementation in Poland and redirected for translation by a native English speaker.

### Development of the first version of the rating reference guide

The rating reference guide Polish version of ICF Rehabilitation Set contains a description how to assess the different ICF categories and how to transfer the assessment results to qualifiers.

Rehabilitation experts have identified the main aspects to consider when rating ICF Body Functions, Activities and Participation, and Environmental Factors. The members of the working group proposed descriptive scales that can be used in practice to rate the categories. The proposed rating system was prepared during a discussion panel based on the applied methods of clinician assessment, cognitive interviews and reviews of available and already verified tools for the assessment of ICF categories. The available guide is also based on the results of the definitions developed by the Japanese Team^19^ and the Italian Team^15^.

### Ethics

This study was approved by the Bioethics Committee of the University of Rzeszów (Resolution No. 33/05/2019). All participants provided written informed consent. All methods were performed in accordance with the relevant guidelines and regulations.

## Results

A Polish version of ICF Rehabilitation Set to assess and verify functioning levels of patients was developed for 29 categories, including 8 categories (B130, B134, B152, B280, B455, B620, B710, B730) from Body Functions, 16 (D230, D240, D410, D415, D420, D450, D465, D510, D530, D540, D550, D570, D640, D710, D850, D920) from Activities and Participation, and 5 (E110, E115, E155, E310, E450) from Environmental Factors. Table 1 shows a comparison of ICF Rehabilitation Set and the initial Polish ICF Rehabilitation Set included the most important ICF categories from the point of view of rehabilitation experts and patients. The main difference between the sets concerns the inclusion of environmental factors in the assessment of patients participating in the rehabilitation process.

**Table 1:** Identification of ICF Rehabilitation Set categories.

ICF components	WHO ICF Rehabilitation Set		Polish version ICF Rehabilitation Set	
	ICF code	ICF categories	ICF code	ICF categories
Body functions	B130	Energy and drive functions	B130	Energy and drive functions
	B134	Sleep functions	B134	Sleep functions
	B152	Emotional functions	B152	Emotional functions
	B280	Sensation of pain	B280	Sensation of pain
	B455	Exercise tolerance functions	B455	Exercise tolerance functions
	B620	Urination functions	B620	Urination functions
	B640	Sexual functions	–	–
	B710	Mobility of joint functions	B710	Mobility of joint functions
	B730	Muscle power functions	B730	Muscle power functions
Activity and participation	D230	Carrying out daily routine	D230	Carrying out daily routine
	D240	Handling stress and other psychological demands	D240	Handling stress and other psychological demands
	D410	Changing basic body position	D410	Changing basic body position
	D415	Maintaining a body position	D415	Maintaining a body position
	D420	Transferring oneself	D420	Transferring oneself
	D450	Walking	D450	Walking
	D455	Moving around	–	–
	D465	Moving around using equipment	D465	Moving around using equipment
	D470	Using transportation	–	–
	D510	Washing oneself	D510	Washing oneself
	D520	Caring of body parts	–	–
	D530	Toileting	D530	Toileting
	D540	Dressing	D540	Dressing
	D550	Eating	D550	Eating
	D570	Looking another one’s health	D570	Looking another one’s health
	D640	Doing housework	D640	Doing housework
	D660	Assisting others	–	–
	D710	Basic interpersonal interactions	D710	Basic interpersonal interactions
	D770	Intimate relationship	–	–
	D850	Remunerative employment	D850	Remunerative employment
	D920	Recreation and leisure	D920	Recreation and leisure
Environment factors	–	–	E110	Products or substances for personal consumption
	–	–	E115	Products and technology for personal use in daily living
	–	–	E155	Design, construction and building products and technology of buildings for private use
	–	–	E310	Immediate family
	–	–	E450	Individual attitudes of health professionals

### Development of simple, intuitive descriptions

As a result of the process of defining ICF categories, 6 initial description proposals were accepted in the first stage, and 15 proposals in the second stage. In the final third voting, the description of the remaining 8 definitions was settled. All participants agreed to the final simple, intuitive descriptions of the ICF categories indicated at the verification stage.

The Polish final version of the simple, intuitive description of the ICF categories was shown on Table 2.

**Table 2:** The Polish version of the simple, intuitive description of the ICF categories.

ICF Code	ICF categories	Simple intuitive descriptions
B130	Energy and drive functions	Mental functions that stimulate self-driven activities in everyday life, e.g. motivation
B134	Sleep functions	Mental functions that are responsible for essential and sufficient sleep
B152	Emotional functions	Mental functions that control emotions, e.g. sadness, joy, fear
B280	Sensation of pain	Feeling of pain
B455	Exercise tolerance functions	Functions of physical capacity necessary to perform activities in everyday life
B620	Urination functions	Functions related to urination
B710	Mobility of joint functions	Functions related to the range and ease of performing movements of the joints
B730	Muscle power functions	Functions related to muscle strength
D230	Carrying out daily routine	Planning and performing daily activities
D240	Handling stress and other psychological demands	Dealing with stress and/or distractions during tasks demanding responsibility
D410	Changing basic body position	Changing the body position, e.g. sitting down, standing up
D415	Maintaining a body position	Maintaining the body position, e.g. standing and/or sitting
D420	Transferring oneself	Transferring oneself, carrying the body, e.g. moving from bed to chair
D450	Walking	Walking on level ground
D465	Moving around using equipment	Moving around with the use of auxiliary devices, e.g. a walker, a walking frame
D510	Washing oneself	Washing oneself, wiping and drying the body
D530	Toileting	Managing urination, defecation and menstruation in everyday life, including washing oneself afterwards
D540	Dressing	Putting on and taking off clothes and shoes
D550	Eating	Eating safely with cutlery
D570	Looking another one’s health	Taking the necessary steps to take care of one’s health
D640	Doing housework	Doing housework in everyday life
D710	Basic interpersonal interactions	Maintaining appropriate interactions with other people, such as being respectful, warm and expressing opinions
D850	Remunerative employment	Engaging in remunerative work
D920	Recreation and leisure	Engaging in recreational activities
E110	Products or substances for personal consumption	Natural or human-made product for human consumption, including food, drugs, vitamins and supplements
E115	Products and technology for personal use in daily living	Equipment, products and technologies used by people in everyday activities, e.g. prostheses, orthoses or home furnishings
E155	Design, construction and building products and technology of buildings for private use	Products and technologies that make it easier for people to move inside and outside buildings, e.g. driveways, elevators, barriers, thresholds, etc.
E310	Immediate family	Immediate family
E450	Individual attitudes of health professionals	Healthcare professionals' attitudes and opinions that influence individual behavior and actions.

### Development of the rating reference guide

The assessment of all three components of the classification was proposed using the same scale described by WHO^7^. For “Body Functions”, a general qualifier that scores the extent of the problem or the extent of the impairment is shown on a scale from 0 to 4 (xxx.0 NO problem; xxx.1 MILD problem; xxx.2 MODERATE problem; xxx.3 SEVERE problem; xxx.4 COMPLETE problem xxx.8 not specified and xxx.9 not applicable).

For “Activities and Participation”, two qualifiers are presented: *capacity* and *performance*. Rating *Capacity* refers to the patient's ability to complete a task or take an action. It should be measured in a unified, standardized environment, so without the use of facilitators and specific barriers. Rating *Performance* refers to the patient's ability to perform a task or take an action in one’s own current environment, thus taking into account the facilitators or barriers posed by the social conditions in which the patient lives. Both qualifiers are scored using the following scale: xxx.0 NO problem; xxx.1 MILD problem; xxx.2 MODERATE problem; xxx.3 SEVERE problem; xxx.4 COMPLETE problem; xxx.8 not specified and xxx.9 not applicable.

The coding of "Environmental Factors" should be done by assessing the patient's perspective. A qualifier assessing environmental factors may indicate a barrier or facilitator. The rating scale is as follows: xxx.0 NO barrier; xxx.1 MILD barrier; xxx.2 MODERATE barrier; xxx.3 SEVERE barrier; xxx.4 COMPLETE barrier; xxx.+ 0 NO facilitator; xxx + 1 MILD facilitator; xxx.+ 2 MODERATE facilitator; xxx.+ 3 SUBSTANTIAL facilitator; xxx.+ 4 COMPLETE facilitator; xxx.8 barrier not specified; xxx.+ 8 unspecified facilitator and xxx.9 not applicable^7^.

For the analysis of limitations in the range of body functions related to B130, B134, B152, B445 and B620, an assessment by means of specially prepared questions and an assessment carried out by healthcare professionals were proposed. The description of the possible limitation makes it possible to qualify the patient's problem for the appropriate qualifier. The assessment of B280 (*Sensation of pain*), B710 (*Mobility of joint functions*) and B730 (*Muscle power functions*) was linked to existing methods and scales commonly used in clinical practice, i.e. pain assessment on the Visual Analog Scale^21^, goniometric assessment of the range motion^22^ and assessment of muscle strength using the Lovett Scale^23^.

In case of activity and participation, D230, D240, D465, D570, D710, D850 and D920 were assessed by means of questions asked by healthcare professionals. The assessment of categories D420 (*Transferring oneself*), D510 (*Washing oneself*), D530 (*Toileting*), D540 (*Dressing*), D550 (*Eating*) was based on the Activity of Daily Living Scale^24^, taking into account the range of patient limitations on a scale from 0 to 4, whereas the assessment of one of the complex daily activities D650 (Doing housework) was presented using the Lawton Instrumental Activities of Daily Living Scale^25^. For the analysis of categories D415 (*Maintaining a body position*) and D410 (*Changing basic body position*), the Berg Balance Scale was used^26^. With reference to mobility assessment D450 (*Walking*), the result of the Short Physical Performance Battery Test was used on the ICF scale^27^.

The assessment guide of environmental categories E110, E115, E155, E310 and E450 focused on the analysis of the existence of barriers and facilitations based on specially developed questions. Tables 3, 4 and 5 presents the rating reference guide Polish version of ICF Rehabilitation Set.

**Table 3:** Rating reference guide Polish version of ICF Rehabilitation Set (Body Functions).

Category	Aspect to be scored	Description of each response option	Translation into ICF Qualifier	Tool
Body functions				
B130	Energy and drive functions	The scope and frequency of the problem, e.g. loss of energy or motivation. Question: How big of a problem is it for the patient to lose the energy and motivation to function?	0: No problem 1: Mild problem: may include problems with energy and motivation functions that do not interfere with the patient's daily activities 2: Moderate problem: may include problems with energy and drive functions that exceeds 1 but remains relatively minor (<50%) 3: Severe problem: may include a significant problem (≧ 50%) in energy and drive functions 4: Complete problem: may include a complete problem with energy and drive functions such as lack of motivation or drive at any time 8: Not specified 9: Not applicable	Self-developed question/Health Professional Assessment (e.g. Psychologist)
B134	Sleep functions	The scope and frequency of the problem, such as lack of sleep or an irregular sleep schedule Question: How big of a problem is it for the patient to lack the sleep or irregular sleep?	0: No problem 1: Mild problem: may include sleep problems that do not affect the patient's daily activities 2: Moderate problem: may have a sleep problem that exceeds 1 but remains relatively minor (<50%) 3: Severe problem: may include a significant sleep problem (≧ 50%) 4: Complete problem: may include complete sleep problem such as unable to sleep or complete day-night reversal every day 8: Not specified 9: Not applicable	Self-developed question/Health Professional Assessment (e.g. Psychologist)
B152	Emotional functions	The scope and frequency of the problem, e.g. loss of emotional control or lack of emotional expression Question: How big of a problem is it for the patient to lose emotional control or lack emotional expression?	0: No problem 1: Mild problem: may include emotional problems that do not affect the patient's daily activities 2: Moderate problem: may include emotional problems that exceed 1 but remain relatively minor (<50%) 3: Severe problem: may include a significant problem (≧ 50%) with emotions 4: Complete problem: may include an complete problem with emotions, such as complete loss of emotional control or being unable to express emotions at any time 8: Not specified 9: Not applicable	Self-developed question/Health Professional Assessment (e.g. Psychologist)
B280	Sensation of pain	The scope and frequency of the problem, e.g. sensation of pain	0: No problem: 0 points 1: Mild problem: 1–2 points 2: Moderate problem: 3–4 points 3: Severe problem: 5–9 points 4: Complete problem: 10 points 8: Not specified 9: Not applicable	Visual Analog Scale Scale/Health Professional Assessment (e.g. Physician; Physiotherapist)
B455	Exercise tolerance functions	The scope and frequency of the problem, such as a decline in respiratory and cardiovascular functions required for daily activities Question: How big of a problem is it for the patient to tolerate the decrease in respiratory and cardiovascular functions required to perform daily activities? Clinical assessment based on observation	0: No problem 1: Mild problem: may include exercise tolerance problems that do not affect the patient's daily activities 2: Moderate problem: may include an exercise tolerance problem that exceeds level 1 but remains relatively minor (<50%) 3: Severe problem: may include a significant problem (≧ 50%) with exercise tolerance 4: Complete problem: may include an complete problem with exercise tolerance, e.g., inability to bear any single daily activity at any time due to problems with the cardio-respiratory system 8: Not specified 9: Not applicable	Self-developed question/Health Professional Assessment (e.g. Physician; Physiotherapist)
B620	Urination functions	The scope and frequency of the problem, such as difficulty urinating or urinary incontinence. Question: How big of a problem is it for the patient to urinate or suffer incontinence?	0: No problem 1: Mild problem: may include urination problems that do not affect the patient's daily activities 2: Moderate problem: may include a urination problem that exceeds 1 but remains relatively minor (<50%) 3: Severe problem: may include a significant problem (≧ 50%) urinating 4: Complete problem: may include an complete problem urinating such as complete urinary retention or continuous urinary incontinence at any time 8: Not specified 9: Not applicable	Self-developed question/Health Professional Assessment (e.g. Physician)
B710	Mobility of joint functions	The scope of the problem, such as joint contracture or restriction of movement, and the percentage of joints with movement problems	0: No problem/normal ranges of motion 1: Mild problem: may include problems with joint mobility functions that do not affect the patient's daily activities (normal ranges of motion within 2/3 of the physiological range, a. the limitation is not greater than 10 degrees) 2: Moderate problem: may include joint mobility problems greater than 1 but remain relatively minor (<50%) (mobility limited to 1/3 of physiological range of motion) 3: Severe problem: may include a significant problem (≧ 50%) with joint mobility (mobility restrictions up to 2/3 of the physiological range of motion) 4: Complete problem: may include an extreme joint mobility problem such as complete joint contracture in all major joints (complete limitation of range of motion) 8: Not specified 9: Not applicable	Movement ranges measured by the goniometer/Health Professional Assessment (e.g. Physician; Physiotherapist)
B730	Muscle power functions	The scope of the problem or limitations related to muscle strength	0: No problem: muscle strength 5 on the Lovett scale 1: Mild problem: may include muscle strength problems that do not affect the patient's daily activities, muscle strength 4 on the Lovett scale 2: Moderate problem: may include a problem with muscle strength function that exceeds 1 but remains relatively minor (<50%), muscle strength 3-2 on the Lovett scale 3: Severe problem: may include a significant problem (≧ 50%) of muscle strength, muscle strength 1 on the Lovett scale 4: Complete problem: may include an extreme muscle strength problem, such as a complete loss of strength in all major muscles, muscle strength 0 on the Lovett scale 8: Not specified 9: Not applicable	Lovett scale/Health Professional Assessment (e.g. Physician; Physiotherapist)

**Table 4:** Rating reference guide Polish version of ICF Rehabilitation Set (Activities & Participation).

Category	Aspect to be scored	Description of each response option	Translation into ICF qualifier	Tool
Activities & participation				
Capacity and performance				
D230	Carrying out daily routine	The scope and frequency of the problem, e.g. difficulties in planning activities. Question: How big of a problem is it for the patient planning everyday activities?	0: No problem 1: Mild problem: patient is acting alone but is weak in their planning/or not active in their implementation 2: Moderate problem: patient performs activities with partial support in planning and carrying out daily activities 3: Severe problem: patient is largely supported in planning and carrying out daily activities 4: Complete problem: patient performs activities with total support/carrying out of the action is impossible. 8: Not specified 9: Not applicable	Self-developed question/Health Professional Assessment (e.g. Physiotherapist)
D240	Handling stress and other psychological demands	The scope and frequency of the problem, e.g. difficulty coping with stress. Question: How big of a problem is it for the patient coping with stress?	0: No problem 1: Mild problem: patient carries out the actions alone but demands advice or encouragement from others to complete tasks 2: Moderate problem: patient carries out activities partially with support and/or instructions from others 3: Severe problem: patient mainly carries out activities with support and/or instruction from others 4: Complete problem: patient performs works with complete support/ carrying out of the action is impossible. 8: Not specified 9: Not applicable	Self-developed question/Health Professional Assessment (e.g. Psychologist)
D415	Maintaining a body position	The scope of the problem related to maintaining a body position, e.g. sitting or standing	0: No problem: patient is able to stand alone and safely for 1 minute 1: Mild problem: patient is able to stand with supervision safely for 1 minute 2: Moderate problem: patient is able to stand unsupported and safely for 30 seconds 3: Severe problem: patient needs help to get into position, but stays there for 15 seconds 4: Complete problem: patient needs help to get into position and does not stay there for 15 seconds 8: Not specified 9: Not applicable	Berg Balance Scale, item 3/Health Professional Assessment (e.g. Physiotherapist)
D410	Changing basic body position	The scope of the problem related to changing the position of the body, e.g. from standing to sitting	0: No problem: patient sits safely with minimal use of hands 1: Mild problem: patient controls sitting down by using his hands 2: Moderate problem: patient uses back of legs against chair to sit down 3: Severe problem: patient sits down but doesn't control movement 4: Complete problem: patient needs assistance to sit, unable to change position by himself. 8: Not specified 9: Not applicable	Berg Balance Scale, item 4/Health Professional Assessment (e.g. Physiotherapist)
D420	Transferring oneself	The scope of the problem related to the movement, e.g. transfer from a bed to a chair	0: No problem: patient is independent 1: Mild problem: patient is slightly dependent 2: Moderate problem: patient needs assistance, but can do some things without assistance 3: Severe problem: patient needs great assistance 4: Complete problem: patient is completely dependent 8: Not specified 9: Not applicable	Activities of Daily Living Scale, item 4/Health Professional Assessment (e.g. Nurse; Physiotherapist)
D450	Walking	Assessment of walking speed at a distance of 4m	0: No problem, trial time 4.81 and less 1: Mild problem: trial time 4.82 - 6.20 s 2: Moderate problem: trial time 6.21–8.70 seconds 3: Severe problem: trial time 8.7 and more 4: Complete problem: participant is unable to perform the test 8: Not specified 9: Not applicable	Short Physical Performance Battery test, item 4/Health Professional Assessment (e.g. Physiotherapist)
D465	Moving around using equipment	The scope of the problem related to moving around with the use of equipment	0: No problem 1: Mild problem: patient walks alone with the use of an orthosis, a walking stick and/or walker/walks alone or under supervision. The patient walks alone with a feeling of difficulty 2: Moderate problem: patient is walking partially with support and/or instructions from others 3: Severe problem: patient mainly walks with support and/or instructions from others 4: Complete problem: patient walks completely with support/or walking is impossible 8: Not specified 9: Not applicable	Health Professional Assessment (e.g. Physiotherapist)
D510	Washing oneself	The scope of the problem related to washing oneself	0: No problem: patient is able to wash oneself 1: Mild problem: patient is able to wash oneself with verbal help and/or supervision 2: Moderate problem: patient is able to wash with some assistance of another person. 3: Severe problem: patient is able to wash oneself with great assistance of another person. 4: Complete problem: patient is unable to wash oneself, needs one person to assist 8: Not specified 9: Not applicable	Activities of Daily Living Scale, item 1/ Health Professional Assessment (e.g. Nurse; Physiotherapist)
D530	Toileting	The scope of the problem related to the use of the toilet	0: No problem: patient is independent (taking off, putting on, getting dressed, maintaining personal hygiene) 1: Mild problem: patient is slightly dependent 2: Moderate problem: patient needs some assistance 3: Severe problem: patient needs great assistance 4: Complete problem: patient is completely dependent 8: Not specified 9: Not applicable	Activities of Daily Living Scale, item 3/Health Professional Assessment (e.g. Nurse; Physiotherapist)
D540	Dressing	The scope of the dressing problem	0: No problem: patient is independent (in fastening buttons, zippers, laces, etc.) 1: Mild problem: patient is slightly dependent 2: Moderate problem: patient needs some assistance 3: Significant: patient needs great assistance 4: Complete problem: patient is completely dependent 8: Not specified 9: Not applicable	Activities of Daily Living Scale, item 2/Health Professional Assessment (e.g. Nurse; Physiotherapist)
D550	Eating	The scope of the eating problem	0: No problem: patient is independent 1: Mild problem: patient is slightly dependent 2: Moderate problem: patient needs some assistance 3: Significant: patient needs great assistance 4: Complete problem: patient is completely dependent 8: Not specified 9: Not applicable	Activities of Daily Living Scale, item 5/Health Professional Assessment (e.g. Nurse; Physiotherapist)
D570	Looking after one’s health	The scope and frequency of the problem, e.g. concern for one's own health Question: How big of a problem is it for the patient concern for one's own health?	0: No problem 1: Mild problem: patient takes care of one’s own health, but requires advice or encouragement from others/takes care of one’s own health with feeling of difficulty 2: Moderate problem: patient takes care of one’s own health with some instruction from others 3: Severe problem: patient cares about his own health mainly with instructions from others 4: Complete problem: patient cares about his own health completely with support and/or taking care of one’s own health is impossible to achieve 8: Not specified 9: Not applicable	Self-developed question/Health Professional Assessment (e.g. Physician)
D640	Doing housework	The scope and frequency of the problem, e.g. doing housework	0: No problem: patient is independent, self-directed 1: Mild problem: patient is slightly dependent 2: Moderate problem: patient needs some assistance 3: Severe problem: patient needs great assistance 4: Complete problem: patient is completely dependent 8: Not specified 9: Not applicable	Lawton Instrumental Activities of Daily Living Scale, item 5/Health Professional Assessment (e.g. Nurse; Physiotherapist)
D710	Basic interpersonal interactions	The scope and frequency of the problem, e.g. basic interpersonal contacts Question: How big of a problem is it for the patient basic interpersonal contacts?	0: No problem 1: Mild problem: patient makes contacts oneself, but requires advice or encouragement from others 2: Moderate problem: patient makes contacts oneself, with some assistance others from others 3: Severe problem: patient interacts, mainly with instructions and great assistance from others 4: Complete problem: patient is unable to make basic interpersonal contacts oneself 8: Not specified 9: Not applicable	Self-developed question/Health Professional Assessment (e.g. Psychologist)
D850	Remunerative employment	The scope and frequency of the problem, e.g. paid employment Question: How big of a problem is it for the patient paid employment?	0: No problem 1: Mild problem: patient is able to paid work, but requires advice or encouragement from others 2: Moderate problem: patient is able to undertake work with some assistance from others 3: Severe problem: patient is mainly able to work with instructions and assistance from others 4: Complete problem: patient is unable to work independently 8: Not specified 9: Not applicable	Self-developed question/Health Professional Assessment (e.g. Physiotherapist)
D920	Recreation and leisure	The scope and frequency of the problem, e.g. the organization of free time Question: How big of a problem is it for the patient organization of free time?	0: No problem 1: Mild problem: patient organizes free time on one's own, but requires advice or encouragement from others 2: Moderate problem: patient organizes free time on one's own with partial instruction from others 3: Severe problem: patient organizes free time mainly with instructions from others 4: Complete problem: patient organizes free time completely with support/or the organization of free time is impossible to achieve 8: Not specified 9: Not applicable	Self-developed question/Health Professional Assessment (e.g. Social Worker; Physiotherapist)

**Table 5:** Rating reference guide Polish version of ICF Rehabilitation Set (Environmental Factors).

Category	Aspect to be scored	Description of each response option	Translation into ICF qualifier	Tool
Environmental factors				
E110	Products and substances for personal consumption - drugs	The scope of facilitators or barriers in the assessment of the use of natural or man-made products or substances for consumption, e.g. drugs Question: Are the medications/supplements used a facilitation or a barrier for the patient’s health?	0: No barrier 1: Mild barrier 2: Moderate barrier 3: Severe barrier 4: Complete barrier + 0: No facilitator + 1: Mild facilitator + 2: Moderate facilitator + 3: Substantial facilitator + 4: Complete facilitator 8: Not specified 9: Not applicable	Self-developed question/Health Professional Assessment (e.g. Social Worker; Physiotherapist)
E115	Products and technology for personal use in daily living	The scope of facilitators or barriers in the assessment of the use of products or technologies for personal use, e.g. orthopaedic supplies Question: Do devices, orthopedic products and technologies (e.g. prostheses, orthoses, etc.) constitute a barrier or a facility for the patient’s health?	0: No barrier 1: Mild barrier 2: Moderate barrier 3: Severe barrier 4: Complete barrier + 0: No facilitator + 1: Mild facilitator + 2: Moderate facilitator + 3: Substantial facilitator + 4: Complete facilitator 8: Not specified 9: Not applicable	Self-developed question/Health Professional Assessment (e.g. Social Worker; Physiotherapist)
E155	Architectural designs, construction and building materials and technologies for buildings for private use	The scope of facilitators or barriers in the field of products and technologies designed inside and outside buildings, e.g. elevator, thresholds, entrance and exit from houses Question: Do elevators, thresholds, entry and exit from the house and buildings constitute a barrier or a facilitation for the patient’s health?	0: No barrier 1: Mild barrier 2: Moderate barrier 3: Severe barrier 4: Complete barrier + 0: No facilitator + 1: Mild facilitator + 2: Moderate facilitator + 3: Substantial facilitator + 4: Complete facilitator 8: Not specified 9: Not applicable	Self-developed question/Health Professional Assessment (e.g. Social Worker; Physiotherapist)
E310	Immediate family	The scope of facilitators or barrier in the field of providing physical or emotional support from the immediate family Question: Are family members and their support a barrier or facilitation for the patient’s health?	0: No barrier 1: Mild barrier 2: Moderate barrier 3: Severe barrier 4: Complete barrier + 0: No facilitator + 1: Mild facilitator + 2: Moderate facilitator + 3: Substantial facilitator + 4: Complete facilitator 8: Not specified 9: Not applicable	Self-developed question/Health Professional Assessment (e.g. Psychologist)
E450	Attitudes of healthcare professionals	The scope of facilitators or barriers to providing physical or emotional support from the attitudes of healthcare professionals Question: Are the opinions and beliefs of healthcare professionals a barrier or a facilitation for the patient’s health?	0: No barrier 1: Mild barrier 2: Moderate barrier 3: Severe barrier 4: Complete barrier + 0: No facilitator + 1: Mild facilitator + 2: Moderate facilitator + 3: Substantial facilitator + 4: Complete facilitator 8: Not specified 9: Not applicable	Self-developed question/Health Professional Assessment (e.g. Social Worker; Physiotherapist)

## Discussion

The use of the ICF in clinical practice requires a precise definition of categories and rating system of qualifiers in a way that will allow for the standardization of the assessment regarding the effects of patients' rehabilitation. The development of the Polish version of ICF Rehabilitation Set included a process of codes verification, development of simple intuitive descriptions for individual ICF categories and a proposal of the rating system of qualifiers to support the implementation of ICF in clinical practice in Poland among patients participating in the rehabilitation process. Prepared descriptions do not exclude linking and using specific measurement and clinical tools in the future to assess a given code. Moreover, it is assumed that the prepared descriptions are used in a compatible manner and linked with data transformed from standardized measurement tools.

Verification and identification of the Polish version of ICF Rehabilitation Set showed 29 ICF categories: 8 categories related to body functions; 16 ones related to activities and participation, and 5 ones related to environmental factors. Comparing these categories to the generic set, the biggest change was the introduction of 5 environmental rating codes. According to the panel of experts, researchers and patients, the following categories were introduced, such as: products and technology for personal use in daily living (e115); immediate family (e310) or individual attitudes of healthcare professionals (e450). These categories are important due to the fact that they allow for the identification of facilitating factors or barriers related to the process of functional rehabilitation of patients^15,28^. Prodinger et al. also proposed the use of a set of 12 environmental categories as a supplement to the assessment of functioning aspects in clinical populations for reporting health conditions, including rehabilitation process^20^. Disability is not only a health problem; it is also the result of the interaction of health status with factors of the living environment and is an integral part of assessment in the field of patient rehabilitation^29^.

The development of a reliable rating tool increases the usefulness of the ICF classification for clinical and statistical purposes. The results on the patient's functioning can be transmitted to other specialists, as well as compared showing the effectiveness of rehabilitation in different institutions or regions^19^. Previous studies on the clinical implementation of the ICF Rehabilitation Set suggested that clinicians were unable to distinguish effectively the line between different qualifiers^30^. Moreover, in the study carried out by Senju et al., experts also indicated that it is difficult to distinguish the difference between mild and moderate ICF problems. The WHO states that a moderate problem ranges from 25 to 49% of problem percentages, whereas a mild problem is from 2 to 24%^31^. The existing explanation of the problem causes some difficulties in assessing patients in clinics^32^.

Therefore, international efforts have been made to develop ICF-based clinical tools. The development of tools includes the development of ICF sets as well as simple intuitive descriptions of ICF categories included in the sets^17^. What is more, some studies regarding the implementation of ICF sets were also carried out, however, the assessment of qualifiers differed from one study to another. The Chinese project used an intuitive rating scale ranging from 0 to 10^33^. The Japanese researchers developed a rating reference guide for the ICF Rehabilitation Set. They developed a rating system with a description for each answer and translated the assessment into ICF qualifiers^19,34^. The weighted kappa coefficient in the field studies was 0.61. The inter-rater reliability test showed moderate to high inter-rater agreement^34^. In the Polish version of the ICF Rehabilitation Set, apart from specially developed questions for the assessment of individual categories, the most frequently used scales in clinical conditions in Poland were also proposed.

One of the most important issues in interdisciplinary communication in healthcare is the use of common terminology^35^. The ICF classification is closely related to the ICD-10. The ICD-10 is a health classification system and etiological framework, while the ICF is used to classify health-related functioning and disability. Thus, the ICD-10 provides information on the diagnosis of the disease, while the ICF offers an additional explanation of how the medical condition affects the functioning of people with various diseases^36^. Combining the ICD-10 reporting with the ICF can improve the quality of data regarding patients collected in the healthcare system.

The World Health Organization and the World Confederation for Physical Therapy proposed the use of the ICF as a universal patient assessment in rehabilitation practice^37^. Therefore, the use of a unified rating system considering ICF qualifiers will be beneficial both in clinical and scientific discussion as well as in statistics^38,39^. The Polish version of ICF Rehabilitation Set proposed by us is a reference framework for the harmonization of existing information on the functioning and disability of people participating in the rehabilitation process.

## Data Availability

The datasets used during the current study are available from the corresponding author on reasonable request.
